# Crude Protein Degradation Kinetics of Selected Tropical Forages in Buffalo Using NorFor In Situ Standards

**DOI:** 10.3390/ani15040585

**Published:** 2025-02-18

**Authors:** Roshan Riaz, Rana Muhammad Bilal, Mahmood Ul Hassan, Massimo Todaro, Riccardo Gannuscio, Fatma Inal, Muhammad Naveed Ul Haque, Muhammad Naeem Tahir

**Affiliations:** 1 Department of Animal Nutrition and Nutritional Diseases, Faculty of Veterinary Medicine, Kafkas University, Kars 36100, Türkiye; roshan.riaz@kafkas.edu.tr; 2 Department of Livestock Management, Faculty of Veterinary and Animal Sciences, The Islamia University of Bahawalpur, Bahawalpur 63100, Pakistan; 3 Department of Animal Nutrition, Cholistan University of Veterinary and Animal Sciences, Bahawalpur 63100, Pakistan; muhammad.bilal@cuvas.edu.pk; 4 Department of Agricultural, Food and Forestry Science (SAAF), University of Palermo, Viale delle Scienze 13, 90128 Palermo, Italy; mahmoodul.hassan@unipa.it (M.U.H.); massimo.todaro@unipa.it (M.T.); riccardo.gannuscio@unipa.it (R.G.); 5 Department of Animal Nutrition and Nutritional Diseases, Faculty of Veterinary Medicine, Selçuk University, Konya 42130, Türkiye; fatmaaksakalinal@gmail.com; 6 Department of Animal Nutrition, Ravi Campus, University of Veterinary and Animal Sciences, Lahore 54000, Pakistan; muhammad.naveed@uvas.edu.pk

**Keywords:** cattle feed, crude protein degradability, tropical forage

## Abstract

This study aimed to evaluate the crude protein (CP) degradation kinetics and degradability (CPD) of cereal and legume fodder species grown at two geographically distant locations. Ten forage species, comprising six cereals (barley, maize, millet, oats, sorghum, and wheat) and four legumes (berseem, jantar, lucerne, and mustard), were evaluated to determine the effects of forage family, species, and location of growth on CP degradation fractions and effective CPD. The forage crops were cultivated under uniform agronomic practices at two distinct agro-ecological locations and were harvested at the booting stage (cereals) and 50% flowering stage (legumes). Dried and ground samples were incubated in the rumen of four Nili-Ravi buffalo fitted with rumen cannula. The incubation periods utilized in the experiment were 0, 4, 8, 16, 24, and 48 h, and a 4 × 2 × 2 split-plot design was employed. The results showed that the CP degradation fractions and CPD were significantly affected by forage family, species, and location of growth. Wide variations in degradation kinetics and degradability existed among and within the cereal and legume fodders, with wheat and jantar ranked at the top. Legume forages had larger soluble fractions, smaller potentially degradable fractions, and rapid rates and extent of degradation of dietary proteins than cereal forages. The cooler climatic conditions at location 2 increased the rapidly degradable protein fraction and overall CPD, whereas the warmer climatic conditions enhanced the slowly degradable protein fractions, thereby reducing the overall protein degradability in tropical forages. It was quite evident that some fodder species, such as maize among the cereal fodders and mustard among the legume fodders, remained quite non-responsive to the effects of the climatic conditions. A moderately positive and linear relationship between the rate of degradation and CPD was established for cereals, whereas a strongly positive and quadratic relationship was established for legume fodders. In conclusion, forage species, family, and location of growth significantly affected the degradation fractions and degradability of tropical cereal and legume fodders.

## Simple Summary

The nutritional needs of livestock are important to the sustainability of food production; however, variations in the quality of forage and environmental conditions can affect the nutritional efficiency of animal feed. This study investigated the variations in crude protein content and its degradability among ten species of cereal and legume fodder grown at two different locations. The feedstuffs comprised six types of cereals and four leguminous crops, all cultivated under similar agronomic practices at each location, and harvested at optimum growth stages. The results of the ruminal protein degradation showed that forage type, species, and geographical location significantly affected protein availability for buffalo. In general, legumes had greater protein availability than cereals and the location of growth affected the overall degradability. Some species were quite less affected by the location of production, while others were more so. The study results will enlighten both farmers and scientists in selecting the appropriate fodder with respect to the climatic conditions to help livestock nutrition and further support sustainable means of farming, which is significant for global food security.

## 1. Introduction

The global livestock industry is facing challenges in meeting the increasing demand for sustainable and high-quality feed resources. This industry has further attracted criticism because of competition among humans and ruminants for resources utilized in their production or for being supplied as food to humans [1]. Ruminants have the ability to convert non-human-utilized agriculture products into high-quality animal products (milk and meat), and forages (cereal and legume) are and should be the main feed resources for ruminants [2]. Although forages are an economical feed source in tropical and subtropical regions, significant fluctuations occur in the quality and availability of these feed resources, ultimately affecting animal performance [3]. Therefore, there is an ultimate need to understand the nutritional dynamics of cereals and legume fodders to optimize their use in meeting the dietary needs of animals.

Among the key nutritional parameters in ruminant nutrition, crude protein (CP) and its degradability are critically important because they govern the nitrogen supply to ruminant microbes, modulating microbial protein synthesis, digestion efficiency, and animal performance [4,5]. The rate and extent of CP degradation in the rumen is critical for formulating the ration to provide the nutritional demands of high-performing animals [6]. Several factors affect the CP degradation kinetics of tropical forages, including agronomic practices, environmental conditions, and forage family and species [7]. Cereal forages show higher degradation due to their lower lignin content, and legume forages possess higher CP levels and rate of degradation but show resistance to fast microbial breakdown due to their high lignin content in the cell wall [5,8]. The regional climate impacts forage growth, its nutritional composition, and the nutrients available for digestion. In addition, variations in the location of the growth environment impact forage CP content and degradation, and protein availability is often reduced from regions with high temperatures and low rainfall [9].

Despite the established utilization of these forages in ruminants, particularly for buffalo, there is a scarcity of data on the CP degradation kinetics of different tropical forage families, species, and geographical locations. In addition, the current ration formulation is also based on the feed evaluation information of either temperate regions or other ruminants from that climate [7,10]. Pakistan, with its diverse agro-ecological regions and varied climatic conditions, has a scarcity of information on fodder nutritional profiles and impacts of the geographic region. There are physiological and metabolic differences between buffalo and other ruminants [11]. These factors ultimately lead to under or over-feeding, resulting in influences on animal performance, economic losses, and the environmental footprint [10]. Therefore, there is a necessity for research to fill this gap to improve feeding strategies for animal production performance and optimize resource utilization efficiency.

This study aimed to determine: (1) the CP degradation parameters of selected tropical forages in Nilli-Ravi buffalo; (2) the influence of forage family, species, and location of growth and their interactions on degradation parameters; and (3) the relationship between the in situ CP degradation rate and crude protein degradability (CPD) for cereal and legume fodders. The findings of this study could contribute to the effective utilization of high-quality feed resources and provide region-specific feeding strategies to increase livestock productive performance and support sustainable agriculture systems in subtropical regions.

## 2. Materials and Methods

### 2.1. Forage Sampling and Processing

**Figure 1:**
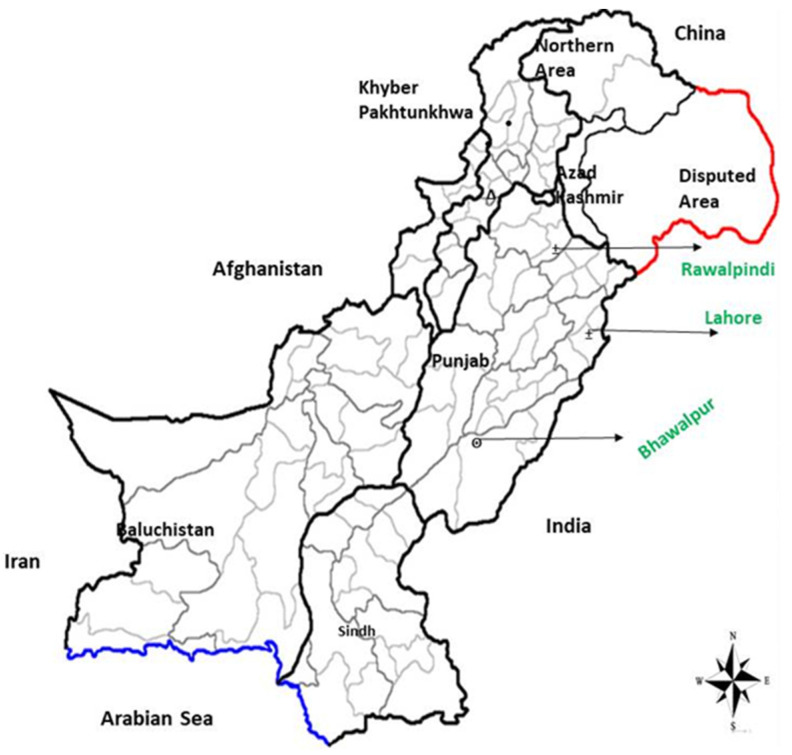
The geographical regions of fodder cultivation.

**Figure 2:**
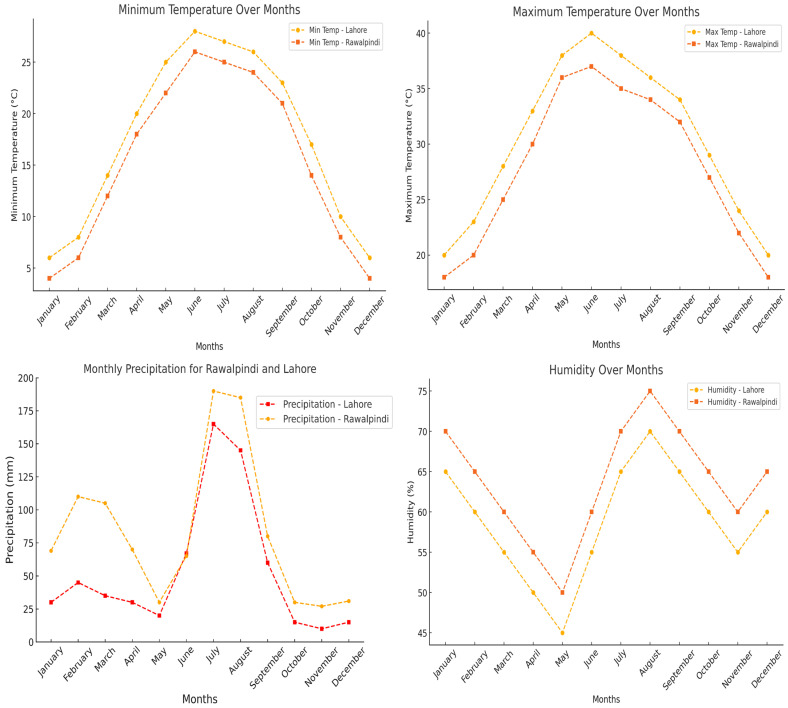

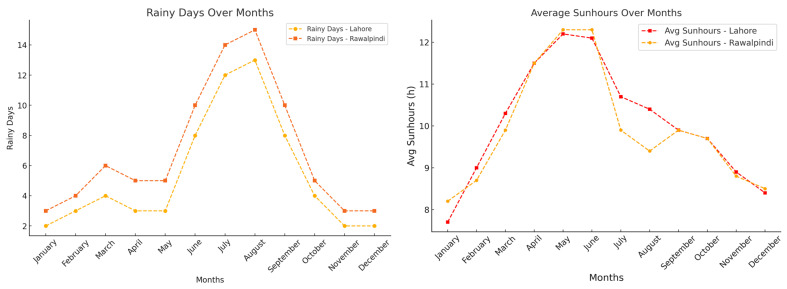
Mean values of climate parameters over months at the growing location.

This study evaluated ten forage species, comprising six cereals and four legumes, across two locations. Summer forages included *Pennisetum glaucum* (millet), *Sorghum bicolor* (Sorghum), *Sesbania bispinosa* (Jantar), and *Zea mays* (maize). Winter forage species consisted of *Avena sativa* (oats), *Brassica napus* (mustard), *Hordeum vulgare* (barley), *Medicago sativa* (lucerne), *Triticum aestivum* (wheat), and *Trifolium alexandrinum* (berseem). Summer crops were planted in mid-March, while winter crops were sown in late November using consistent, locally recommended agronomic practices and geographical production conditions. Each species was cultivated at two different locations: Lahore (31.55° N, 74.35° E; Location 1) for the central region and Rawalpindi (33.598° N, 73.04° E; Location 2) for the northern region of Punjab Province, Pakistan (Figure 1). Within each location, three different plots were cultivated ~100 m apart for statistical sample replication. The geographic climatic conditions and soil profiles of the production sites are presented in Table 1 and Figure 2.

From each site, ~10 kg of herbage was collected for each forage type from three replicate plots. The samples of each forage type collected from three replicated plots at a single location were pooled in order to obtain a more representative sample with as little variation in chemical composition as possible. This pooled sample from each location was further utilized for chemical (two statistical replicates) and in situ analysis (two experimental replicates). To ensure the optimal growth stage for analysis, the cereal and legume forages were harvested at the booting and 50% flowering stages, respectively. The freshly collected herbage was chopped to roughly 20 mm lengths using a chaff cutter (Toka 510, Patiala Agri-Industries, Faisalabad, Pakistan). Following chopping, the samples were air-dried under shade for three to seven days to achieve the recommended moisture content. After drying, the samples were ground using a hammer mill (POLYMIX PX-MFC, Kinematica GmBH, Eschbach, Germany) to two specific particle sizes: 1 mm for chemical analysis and 2 mm for in situ digestion kinetics experiment. The processed samples were stored at room temperature in small plastic containers until further analysis.

### 2.2. Management of Experimental Animals

A total of four rumen-cannulated (Bar Diamond, Parma, ID, USA), Nili-Ravi buffalo (average live weight [LW] = 529 ± 33.4 kg, age = 2635 ± 49.5 days, and parity 3) were used in this study. All animals received a standardized diet formulated to meet maintenance requirements according to the NorFor standards for rumen-cannulated animals [11]. The diet consisted of freshly chopped forage, commercial concentrate, lucerne hay, and cotton seed cake with a forage to concentrate ratio of 85:15. The composition of the diet is presented in Table 2. The animals were individually housed in 2 × 2.5 m stalls, fed separately, and had access to fresh water at all times.

### 2.3. In Situ Incubation and Degradation Profiles

The CP degradation characteristics of the forage samples were studied according to NorFor guidelines using an in situ incubation technique [12]. Approximately 1 g of each sample was incubated under standard conditions of 15 mg per cm² density relative to the bag surface area. The samples were then enclosed in bags and adhesive-sealed in Dacron-type polyester pouches (Sefar AG, Heiden, Switzerland), measuring 11 × 8.5 cm (effective dimensions of 10 × 7.5 cm). The incubation bags had 33 µm pores and were made of PES material (140/37), with 25% free space for optimum entry of rumen fluid. Using the all-in system, the bags with the samples were simultaneously placed into the rumen of each rumen-cannulated animal and removed after 0, 4, 8, 16, 24, and 48 h. The bags, once removed, were washed with tap water and stored at −18 °C until the final incubation time was completed. After the entire incubation batch, the bags were thawed and washed twice with tap water at 25 °C. At the completion of each batch, the intact samples and residues were analyzed for CP content using the Dumas method [13].

### 2.4. Chemical Analyses

Samples of the diet and individual feeds were collected after a fortnight and pooled against each feed. For freshly chopped forage, the dry matter (DM) and ether extract (EE) contents were determined in accordance with AOAC guidelines [13]. The DM content of the dry feed was determined by drying the feed at 105 °C for 16 h in order to remove almost all moisture and to establish a better comparison of the chemical analyses among various forage species. The ash content was measured by the incineration of samples at 525 °C for 6 h (method 923.03). The ether extract (EE) content was evaluated through a 6 h extraction utilizing petroleum ether (method 7.062). The amylase-treated neutral detergent fiber (aNDF) content was determined utilizing an amylase-treated procedure based on the method of Van Soest et al. [14], as modified by Mertens et al. [15]. The non-fiber carbohydrate content was calculated as follows:

$$\left[NFC\;\left(\frac{g}{kg}DM\right)=1000-\left(CP+EE+aNDF+ash\right)\right]$$

### 2.5. Data Analysis and Curve Fitting

The in situ degradation data were categorized into two main components: the washable fraction (*a*), which comprised the initial loss after washing at 0 h, and the non-washable fraction. The latter was further subdivided into a potentially degradable fraction (*b*) and an indigestible fraction, representative of the degradation and residues obtained at the final incubation time point, respectively, using the procedures of Ørskov and McDonald [16]. A first-order kinetic model using Table Curve 2D software (version 5.0, Systat Software Inc., San Jose, CA, USA) was fitted to the degradation data. This model assumes that the conditions of degradation and passage are in a steady state and is expressed as follows:

Yt=a+b(1−exp⁡(−Kdt))

In this equation, *Y*_t_ represents the gradient fraction at time *t*, *K_d_* is the fractional degradation rate of fraction *b*, and *t* denotes the incubation time in hours. The effective ruminal CPD was determined using Ørskov and McDonald’s model, which assumes a fractional passage rate (*K_p_*) of 0.05/h for forages, equivalent to a 20 h rumen retention time [16]. This method facilitated the estimation of effective degradability under assumed rumen kinetics for the forage samples:

$$CPD=\alpha +\left[b\times \frac{Kd}{Kd+Kp\;}\right]$$

### 2.6. Statistical Analysis

Statistical analyses were performed using the GLM procedure in SAS^®^ software (Statistical Analyses Software, version 9.2). The data on the in situ parameters were analyzed considering each buffalo as an experimental replicate using the following ANOVA model:

Y_ijk_ = µ + S(F)_ij_ +F_j_ + L_k_ + (F × L)_jk_ + ε_ijk_

in which Y_ijk_ is the dependent variable, µ is the overall mean, S_i_ shows the effect of the ith forage species and F_j_ shows the effect of the jth forage family (cereal vs. legume), L_k_ shows the effect of the kth location (Lahore vs. Rawalpindi), (F × L)_jk_ is the interaction between the ith forage species and kth location, and ε_ijk_ is the residual error. The least-squares means were compared using *p*-values adjusted according to the Tukey–Kramer multiple comparison test. Results were considered significant when *p* ≤ 0.05 and are presented as the least square mean with the standard error of the mean.

## 3. Results

### 3.1. Chemical Composition of Forage Family

The chemical composition of the forage species is presented in Table 3. The CP values were consistently lower in the cereals compared to the legumes, ranging from 50.6 g/kg DM (sorghum) to 74.5 g/kg DM (wheat) for cereal forages and from 110.3 g/kg DM (mustard) to 135.5 g/kg DM (lucerne) for legume forages. The CP values did not differ across Location 1 and Location 2, as shown by Tahir et al. [7].

### 3.2. Degradation Parameters and Effective CPD by Forage Species and Family

The in situ CP degradation kinetics and effective CPD for cereal and legume fodders are presented in Table 4. Significant differences in degradation kinetics and CPD were observed across cereal and legume species (*p* < 0.001). Wheat exhibited the highest washable fraction (<0.001) and showed the highest CPD value at 48 h. By contrast, sorghum demonstrated the lowest fraction *a* and CPD values and the highest potentially degradable fraction (fraction *b*). Among the legume species, mustard showed the highest fraction *a* value, and the highest fraction *b* value was found in lucerne. The CPD values were significantly higher (*p* < 0.001) in jantar and mustard than in lucerne and berseem. The fraction *c* and *K_d_* values tended to differ across forage species (*p* < 0.10), and the highest *K_d_* value was found in jantar and the lowest was found in sorghum.

The forage family showed a significant effect on the CP degradation kinetics and CPD (*p* < 0.001). The fraction *a*, *K_d_*, and CPD mean values of the legume fodders were significantly higher than those of the cereal fodders. The legumes were characterized by lower fraction *b* values compared to the cereal fodders.

**Figure 3:**
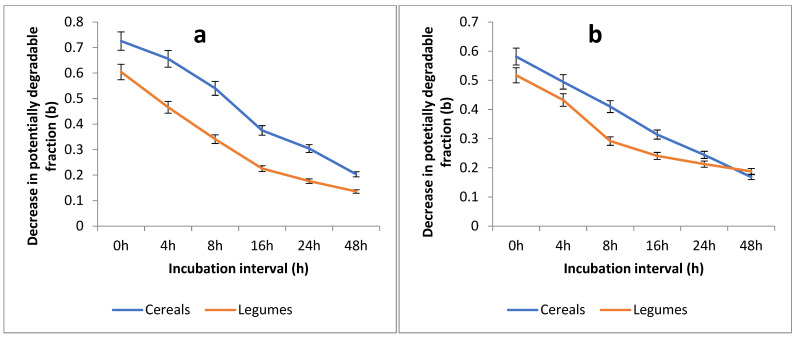
(**a**,**b**) The decrease in potentially degradable degradation fraction *b* of various fodder species averaged against cereal and legume fodders collected from Location 1 (Lahore; (**a**)) and Location 2 (Rawalpindi; (**b**)).

The decreases in fraction *b* values (degradation curves) of the cereal and legume fodders (averaged against respective forage species) with passage of time at both locations are presented at Figure 3a,b. At both locations, legumes were degraded at faster rates than cereals up to 24 h of incubation, pointing to faster disappearance of fraction *b*. It is evident that the two curves are closer to each other at Location 2, whereas they are apart from each other at Location 1. It is evident that the two curves move apart at earlier hours of incubation (up to 24 h); however, the two curves move closer to each other at later incubation intervals.

### 3.3. Degradation Parameters and Effective CPD by Location

The effect of the location of growth on the degradation parameters and effective CPD of cereals and legumes are summarized in Table 4. The location of growth significantly affected all degradation fractions and effective CPD in both cereal and legume forages (*p* < 0.05).

A family by growing location interaction tended to change fraction *a* and CPD for all forages (*p* < 0.10), but it significantly affected fraction *c* (*p* < 0.001). It tended to increase fraction *a* and CPD values in cereals (*p* < 0.10) but had no effect on fraction *a* and CPD values in legumes (*p* > 0.10). This interaction also caused an increase in the value of fraction *c* of legumes and a decrease in the same fraction of cereals at Location 2 compared to Location 1. This interaction did not change the values of fraction *b* and *K_d_* across forage families and/or location of growth (*p* > 0.10).

**Figure 4:**
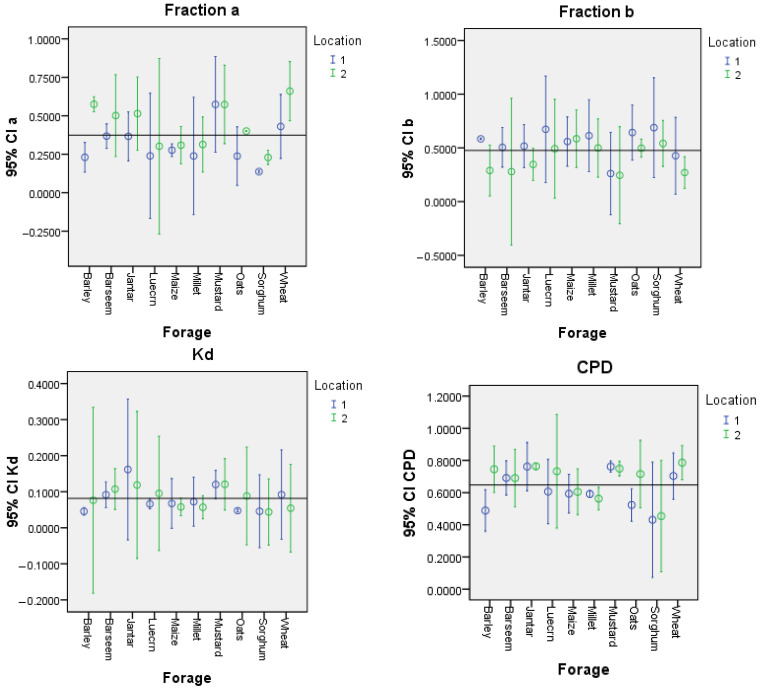
Marginal means with 95% confidence interval (CI) bars of various crude protein fractions of cereal and legume fodders collected from two locations (Location 1 = Lahore, Location 2 = Rawalpindi).

The marginal means with 95% confidence interval bars of species by location of growth interactions for forage species are presented in Figure 4. The location effects were evident with different species responses. The proportion of fraction *a* was increased whereas that of fraction *b* dropped significantly for all species except maize and mustard at Location 2 compared to Location 1. The value of *K_d_* increased for all forage species, except for jantar, millet, and wheat where it dropped at Location 2 compared to Location 1, and it remained unchanged for mustard and millet. The CPD value increased for all forage species, except for millet where it declined, and it remained unchanged for berseem and jantar at Location 2.

### 3.4. Relationship Between CPD and Rate of Degradation

**Figure 5:**
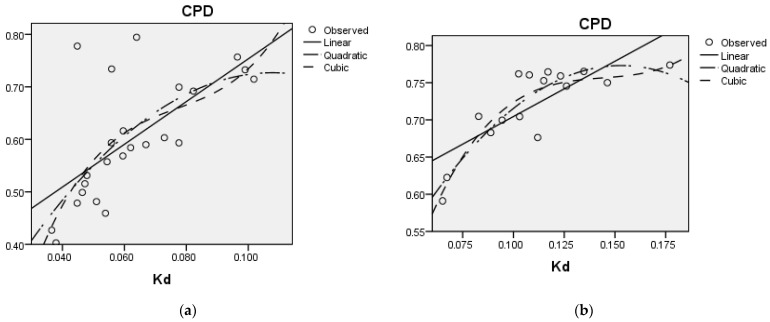
(**a**,**b**) The relationship between rate of degradation (*K_d_*/h) and crude protein degradation (CPD) of cereal fodders (**a**) and legume fodders (**b**) collected from two locations.

Figure 5a,b show the relationships between *K_d_* and CPD for the cereal and legume fodders considered summed across two locations, described using various curve fit models. The data on the regression models are presented in Table 5. The relationship between *K_d_* and CPD was moderately positive (R^2^ = 0.44, SE of estimate = 0.088; linear models) for cereal fodders and it did not change when quadratic or cubic models were applied. Figure 5b shows better values for legume fodders (R^2^ = 0.60, SE of estimate = 0.035; linear models) between *K_d_* and CPD, and the fit was improved when quadratic or cubic models were applied.

## 4. Discussion

### 4.1. Chemical Composition of Forage Family

The CP content exhibited significant variations among forage families and species. The CP values for cereals were comparable to those reported by Sarwar et al. [18], while legumes generally demonstrated lower CP content compared to the values presented by other researchers for tropical legume forages e.g., Singh et al. [19], and higher values in comparison to those of Rehman et al. [20]. Studies have demonstrated that dietary CP content influences the in situ degradation kinetics of crude protein by modulating the solubility and degradability of protein fractions, rumen microbial activity, and passage rate of feed, all of which collectively determine CP degradation kinetics [21,22].

### 4.2. Degradation Parameters and Effective CPD by Forage Species and Family

The development of a comprehensive database on the ruminal protein degradation kinetics of commonly utilized feed sources is essential for optimizing ruminant nutrition. This study presents a dataset on the protein degradation characteristics of forage species prevalent in tropical and subtropical regions, thereby addressing the increasing demand for detailed information on ruminal degradation kinetics of protein.

The results of the CP degradation characteristics demonstrated significant differences in the CP degradation kinetics and CPD between cereal and legume fodders. Among the cereals, wheat exhibited the highest CPD value with a high washable fraction. However, sorghum demonstrated the lowest CPD value and the highest potentially degradable fraction. The legumes, on the other hand, showed more pronounced variability in the degradation parameters. Mustard had a high fraction *a* value, jantar showed a high *K_d_* value, and lucerne displayed a low CPD value with the lowest *K_d_* value. The findings of this study regarding protein fractions *a* and *b* are consistent with the results of Das et al. [23]. The CP degradability results of the cereals were also comparable to those reported by Kaithwas et al. [24]. Singh et al. [25] also identified slow degradation in sorghum. However, the results of wheat degradation in the present study are comparable to those of Islas and Soto-Navarro [26]. Conversely, the degradation parameters observed for the legumes in this study differed from those reported for lucerne by Aufrère et al. [27]. These findings highlight the potential influence of species-specific characteristics on the degradation kinetics of proteins in ruminants.

Legume forages demonstrated superiority over cereals in terms of degradation kinetics and effective CPD, confirming their enhanced protein availability. The steeper degradation curves for legumes, indicative of rapid initial degradation, suggested that they facilitate more expeditious nutrient release. By contrast, cereals exhibited slower CP degradation rates. These results align with previous studies that have reported higher levels of rapidly degradable fractions and CPD in legumes compared to cereals [24,28]. Specific leguminous plants, such as lucerne and berseem, have been shown to yield significant crude protein levels [19,29]. Valderrama et al. [22] also found that legumes demonstrated the highest fraction *a* and CPD values with quite variable degradation rates. This distinction is significant for ruminants, as higher CP degradability in the rumen leads to increased ammonia nitrogen availability [28]. The rapid degradation of crude protein in these legumes is attributed to their structural composition, which supports accelerated microbial activity within the rumen [30]. By contrast, cereal fodders generally exhibit lower protein contents but are frequently characterized by higher carbohydrate levels (aNDF) [30], which can influence the fermentation process within the rumen [24,31]. The slower protein degradation rate of cereals can limit their effectiveness as protein sources when used in isolation; however, grasses exhibit different degradation kinetics from grains due to their fiber-bound proteins and structural composition [32]. Therefore, the balance in CP degradation kinetics is crucial for ruminant nutrition.

### 4.3. Degradation Parameters and Effective CPD by Location

In the present study, the significant effects of the forage growing location on the CP degradation fractions and CPD of cereals and legumes highlight the influence of growing location on forage quality. The least square means of the cereals demonstrated higher fraction *a* values (49% increase) and lower fraction *b* values (13% decrease) at Location 2. Almost similar trends in the location of growth effects were found for various legume fodders for the same parameters (13% increase in fraction *a* value and 23% decrease in fraction *b* value). These results demonstrated that both rapidly soluble and slowly degradable fractions of CP are affected by the climatic conditions, regardless of the origin of the plant family. Cooler climatic conditions increased the rapidly degradable protein fraction and overall protein degradability, whereas warmer climatic conditions enhanced the slowly degradable protein fractions, thereby reducing the overall protein degradability in the tropical forages in the present study. It is worth noting that the climatic conditions had almost no effect on the rate of degradation of fraction *b*, regardless of forage family. It is quite evident that some fodder species, such as maize and sorghum among the cereal fodders and mustard among the legume fodders, remained quite non-responsive to the effects of the climatic conditions and showed quite comparable CPD values at the two locations (Figure 4). These variations in digestibility and degradation behavior can be attributed to the genetic and compositional traits of the crops, which enable adaptation to environmental conditions. Maize and sorghum are contrasting species among the C4 cereals that show adaptations to abiotic stress by reducing the leaf area, transpiration restriction, water use efficiency, germ plasm variability, drought and heat tolerance, and efficient nutrient intake, thus preserving the nutritional value in arid, semi-arid, and tropical regions [33,34,35]. Similarly, a study on the environment effects on mustard showed higher adaptability of mustard to environment-associated stress, such as high temperature, low rainfall, and late sowing [36]. It is further added that the magnitude of the response to location varied among forage species, with certain species exhibiting greater responsiveness, while others demonstrated increased resistance to location effects (Figure 4). This location-based variability aligns with previous studies that have emphasized geographical influences on CP chemical composition and degradation characteristics [37,38,39]. Gruber et al. [38] further elaborated that fractions *a* and *b* were significantly influenced by the location of growth. This agrees with the results of a previous study [39] that indicated the predominant role of genotype × environment interactions in determining the chemical composition and digestibility of grain. The study’s results further demonstrated that the location of growth potentially induces variations in nutrient profiles, affecting both carbohydrate and protein fractions. These variations underscore the importance of site-specific assessment of forage quality, especially in species with wide environmental adaptability.

The geographic location determines the temperature, precipitation, and soil quality, which subsequently affect forage composition and nutritional value. Altitude has been demonstrated to significantly impact soil properties, species composition, and forage production, thereby influencing variability in CP degradability [40]. Forages cultivated at higher altitudes, such as Location 2, experience cooler temperatures and slower plant maturation, which promotes an accumulation of digestible carbohydrates and higher crude protein concentrations, thus elucidating the superior CP degradability values observed at Location 2 compared to Location 1, as reported by Dongdong et al. [41]. Altitude also influences forage quality by altering soil organic carbon and environmental factors such as temperature and precipitation. Tahir et al. [2] reported that variable climatic conditions differing in temperature and precipitation significantly affected fiber concentrations and degradability, consequently affecting forage quality. Similarly, another study showed that climate change, characterized by elevated temperatures and altered precipitation patterns, can negatively impact forage production, reducing CP availability [42].

Temperature variations across the location of growth can affect fodder digestibility by modulating the nutritional composition of forages. Buxton [43] reported that low temperatures slow down plant development, which promotes the accumulation of soluble sugars and enhances digestibility. Cooler climates also reduce the deposition of structural carbohydrates, maintaining higher CP solubility. Conversely, elevated temperatures, as at Location 1, accelerate lignification and increase the quantity of structural carbohydrates, which diminishes fodder digestibility even when the same plant species are involved [44]. Furthermore, the leaf-to-stem ratio, a critical factor in forage quality, is reduced by higher temperatures, as leaves typically possess a higher protein content than stems [41]. These findings are consistent with those of Niklas and Fernandes et al. [37,45], who observed that, at elevated temperatures, tissues with physiological activity exhibited lower levels of CP and increased concentrations of crude fiber. The implications of these structural and biochemical alterations manifested in reduced degradation kinetics under the high-temperature conditions of Location 1.

At Location 2, moderate precipitation facilitated higher CP degradation fractions, presumably due to optimal water availability for plant growth and nutrient uptake. These observations align with those of Li et al. [46], who reported that legumes exhibited higher CP levels under moderate precipitation conditions, while cereals demonstrated increased susceptibility to reduced digestibility under water stress. Consequently, at Location 1, reduced precipitation intensified lignification and increased the accumulation of structural carbohydrates, thereby reducing CP solubility and degradation kinetics [44]. Nevertheless, species-specific responses to precipitation were observed, with legumes demonstrating insensitivity compared to cereals. These plant species exhibited resilience to low or erratic rainfall in such environments.

The degradation kinetics of CP in the rumen varied depending on the forage family in conjunction with the effect of location of growth. Usually, legumes exhibited higher variability in protein degradable fractions (fraction *a*, fraction *c*, and CPD; Table 4 and Figure 3a,b) compared to cereals [47]. In legumes, more soluble and rapidly degradable protein fractions are due to structural differences in their cell walls and nitrogen composition; however, these fractions are vulnerable to changes in climatic conditions [48]. The legume forages showed more sensitivity to environmental factors and changes in chemical composition may occur. Higher temperature and low precipitation result in more proteins complexed to lignin, complexes formed between tannins and proteins, and Maillard reaction products [49]. These components are typically neither degradable in the rumen nor digestible in the post-ruminal tract [19]. Species-based seasonal variations also occur in the amino acid composition, which may influence ruminal digestibility [50]. In addition, the molecular structure characteristics of proteins exhibit significant correlations with protein solubility, rumen degradation, and intestinal digestibility of proteins [51]. Overall, these changes cause modifications in fodder quality and changes in available CP content and ruminal CP degradability.

### 4.4. Relationship Between CPD and Rate of Degradation

This study revealed a strong and statistically significant relationship between *K_d_* and CPD, indicating that increased protein degradation rates are closely associated with higher protein degradability in various fodders. This strong relationship supports the hypothesis that rapid degradation of dietary CP enhances protein solubilization, absorption, and synthesis of microbial protein within the rumen. The marginally higher R^2^ values observed for legumes suggested that the greater variability in the *K_d_* values of cereals resulted in a weaker association between the degradation rate and protein degradability. The strong *K_d_* and CPD relationship observed for legumes in the present study aligns with previous studies highlighting the crucial role of *K_d_* in determining degradability [22]. However, it should be noted that this relationship is quadratic and the marginal response of CPD to an increase in *K_d_* diminishes as the value of *K_d_* reaches beyond a certain level. The study also showed that the CPD of forage crops increased with an increase in fraction *a* and decrease in fraction *b* (observation only, analyses not performed).

## 5. Conclusions

Crude protein fractioning from tropical forages is significantly affected by the forage species, family, and the location of growth. The results showed considerable variability in degradation kinetics and CPD among cereal and legume fodders, with wheat and jantar ranked at the top. Legume forages had larger soluble fractions, smaller potentially degradable fractions, and rapid rates and extent of degradation of dietary proteins than cereal forages in the rumen of buffalo. Cooler climatic conditions increase the rapidly degradable protein fraction and overall protein degradability, whereas warmer climatic conditions enhance the slowly degradable protein fraction, thereby reducing the overall protein degradability in tropical forages. A strong positive relationship between *K_d_* and CPD suggests that *K_d_* can be a significant determinant in predicting the degradation of forage plants within the rumen. The CP degradation kinetics of cereal and legume fodders reported in this study can be utilized for optimal diet formulation.

## Figures and Tables

**Table 1:** Growing location and soil profile characteristics [2].

Parameter	OM (%)	Soil pH	Available K (mg/kg)	Available N (mg/kg)	Available P (mg/kg)	EC (dS/m)	Soil Texture	Ht (m)	PP (mm)	Topography
Lahore	0.87	7.8	158	0.054	14.3	0.67	Clay loam	210	1000	Flat and slope toward south
Rawalpindi	1.42	7.9	112	0.062	11.12	0.36	Clay loam	530	1300	Hilly sub-terrain

OM = organic matter, EC = electrical conductivity, Ht = height from sea level, PP = precipitation (annual).

**Table 2:** Ingredients and chemical composition of the diet offered to rumen-cannulated animals.

No. of Samples = 5, No. of Statistical Replicates = 2, Total No. of Observations per Feed = 10	
	**Mean ± SD**
Ingredients (g/kg on fresh matter)	
Sorghum forage	807.5 ± 10
Lucerne hay	141.5 ± 3
Cotton seed cake	15.0 ± 1
Concentrate mixture	35.5
Forage to concentrate ratio (on DM basis)	85:15
Chemical composition (g/kg DM)	
DM (as fed)	331 ± 10
aNDF	575 ± 12
CP	59.0 ± 3
EE	17.5 ± 2
NFC	234 ± 7
Ash	113 ± 6

DM = dry matter; aNDF = amylase-treated neutral detergent fiber; CP = crude protein; EE = ether extract; NFC = non-fiber carbohydrates. Adapted from Tahir et al. [7].

**Table 3:** Effect of forage family, forage species, and location of growth on the chemical composition. The *p*-values are presented in this table, where *p* ≤ 0.05 shows a significant effect. The values are presented as the least square mean (forage species) with the standard error of the mean unless otherwise stated.

Factor		DMfresh	DMdry	aNDF	CP	EE	Ash	NFC
No. of Samples = 2, No. of Statistical Replicates = 2, Total No. of Observations/Feed = 4								
**Species (Family)**								
Cereal fodders								
Barley		15.4 ^d^	95.3 ^bc^	639.0 ^a^	46.8 ^e^	14.3 ^bcd^	119.6 ^bcd^	180.4 ^b^
Maize		19.6 ^e^	95.2 ^bc^	621.1 ^a^	55.8 ^e^	9.0 ^d^	94.9 ^de^	219.2 ^b^
Millet		19.8 ^e^	94.4 ^ab^	611.3 ^a^	71.2 ^d^	15.3 ^bc^	104.5 ^cde^	197.8 ^b^
Oat		16.5 ^d^	95.4 ^bc^	612.8 ^a^	52.8 ^e^	17.6 ^bc^	91.8 ^de^	225.0 ^b^
Sorghum		21.9 ^f^	94.0 ^a^	607.9 ^a^	53.3 ^e^	17.3 ^bc^	86.7 ^e^	234.8 ^b^
Wheat		15.4 ^b^	95.9 ^cd^	527.7 ^b^	76.8 ^d^	15.5 ^bc^	146.3 ^b^	233.7 ^b^
Legume fodders								
Berseem		8.3 ^a^	96.8 ^d^	429.7 ^c^	110.3 ^b^	13.5 ^cd^	204.6 ^a^	241.9 ^b^
Jantar		16.8 ^d^	94.4 ^ab^	480.9 ^bc^	93.7 ^c^	20.2 ^b^	79.0 ^e^	326.3 ^a^
Lucerne		13.2 ^bc^	95.2 ^bc^	183.6 ^bc^	131.5 ^a^	14.7 ^bcd^	148.0 ^b^	222.1 ^b^
Mustard		14.9 ^cd^	95.5 ^bc^	529.5 ^b^	80.4 ^cd^	28.1 ^a^	127.3 ^bc^	234.7 ^b^
SEM		0.25	0.44	16.28	4.26	1.73	8.24	17.70
**Family**								
Cereals		17.5	95.0	603.3 ^a^	59.5 ^b^	14.8 ^b^	102.3 ^b^	215.1 ^b^
Legumes		13.3	95.5	480.9 ^b^	104.0 ^a^	19.1 ^a^	139.7 ^a^	256.2 ^a^
SEM		0.22	0.12	7.39	1.94	0.77	3.74	8.03
**Location**								
Lahore		17.0	95.1	563.8 ^a^	80.8	17.1	124.3	213.9 ^b^
Rawalpindi		14.6	95.3	520.4 ^b^	82.6	16.8	122.7	247.5 ^a^
SEM		0.19	0.11	7.43	1.94	0.79	3.76	8.08
Family*Location								
Cereals	Lahore	19.2	95.2	644.4 ^b^	54.7 ^c^	14.7	105.3	180.9
Cereals	Rawalpindi	15.9	94.9	562.2 ^c^	64.2 ^b^	14.9	109.3	249.3
Legumes	Lahore	13.9	95.4	483.3 ^a^	106.9 ^a^	19.6	143.4	246.8
Legumes	Rawalpindi	12.8	95.6	478.6 ^a^	101.1 ^a^	18.7	136.0	265.7
SEM		0.25	0.17	10.46	2.74	1.06	5.00	11.66
***p*-value**								
Species (Family)		0.001	0.001	0.002	0.001	0.001	0.001	0.005
Family*Location		0.001	0.345	0.001	0.010	0.576	0.292	0.039
Family		0.001	0.444	0.001	0.001	0.001	0.001	0.001
Location		0.001	0.617	0.001	0.515	0.764	0.755	0.007

DMfresh = dry matter reported at 60 °C for 48 h; DMdry = dry matter reported at 105 °C for 16 h; aNDF = amylase-treated neutral detergent fiber; CP = crude protein; EE = ether extract; NFC = non-fiber carbohydrates. Different lower-case superscripts in a column indicate a significant difference (*p* < 0.01).

**Table 4:** Effect of forage family, forage species, and location of growth on in situ crude protein degradation kinetics and effective degradability of cereal and legume fodders sown in Lahore and Rawalpindi. The *p*-values are presented in this table, where *p* ≤ 0.05 shows a significant effect. The values are presented as the least square mean (forage species) with the standard error of the mean unless otherwise stated.

		**In situ Parameters ^1^**				
**Factor**		***a***	***b***	***c***	***K_d_***	**CPD** **^2^**
No. of Samples = 2, No. of Statistical Replicates = 2, Total No. of Observations/Feed/Incubation Interval = 4						
**Species (Family)**						
Cereal fodders						
Barley		0.403 ^b^	0.437 ^b^	0.160 ^abc^	0.061 ^cd^	0.617 ^bc^
Maize		0.293 ^c^	0.572 ^a^	0.135 ^b^	0.063 ^cd^	0.599 ^bc^
Millet		0.276 ^c^	0.556 ^a^	0.167 ^ab^	0.065 ^cd^	0.577 ^c^
Oats		0.319 ^c^	0.570 ^a^	0.110 ^c^	0.068 ^bcd^	0.620 ^bc^
Sorghum		0.183 ^d^	0.615 ^a^	0.201 ^a^	0.045 ^d^	0.443 ^d^
Wheat		0.545 ^a^	0.349 ^b^	0.106 ^c^	0.073 ^bc^	0.745 ^a^
Legume fodders						
Berseem		0.435 ^b^	0.392 ^b^	0.173 ^ab^	0.099 ^b^	0.691 ^ab^
Jantar		0.440 ^b^	0.432 ^b^	0.128 ^b^	0.140 ^a^	0.763 ^a^
Lucerne		0.271 ^c^	0.583 ^a^	0.147 ^abc^	0.081 ^b^	0.670 ^ab^
Mustard		0.574 ^a^	0.253 ^c^	0.173 ^ab^	0.120 ^a^	0.755 ^a^
SEM		0.029	0.031	0.016	0.009	0.027
**Family**						
Cereals		0.336 ^b^	0.516 ^a^	0.146	0.062 ^b^	0.600 ^b^
Legumes		0.429 ^a^	0.414 ^b^	0.155	0.110 ^a^	0.719 ^a^
SEM		0.014	0.015	0.008	0.004	0.013
**Location**						
Lahore		0.323 ^b^	0.537 ^a^	0.139	0.086	0.630 ^b^
Rawalpindi		0.444 ^a^	0.394 ^b^	0.162	0.087	0.689 ^a^
SEM		0.013	0.014	0.008	0.004	0.012
Family*Location						
Cereals	Lahore	0.259	0.586	0.156 ^ab^	0.062	0.555
Cereals	Rawalpindi	0.415	0.447	0.138 ^b^	0.063	0.645
Legumes	Lahore	0.387	0.489	0.124 ^b^	0.110	0.705
Legumes	Rawalpindi	0.473	0.341	0.186 ^a^	0.110	0.734
SEM		0.019	0.020	0.011	0.006	0.017
***p*-value**						
Species (Family)		0.001	0.001	0.004	0.010	0.001
Family		0.001	0.001	0.441	0.001	0.001
Location		0.001	0.001	0.044	0.895	0.002
Family*Location		0.073	0.807	0.001	0.971	0.095

*a* = washable fraction representing the portion of CP that had disappeared at time 0; *b* = potentially degradable CP fraction; *c* = indigestible fraction (1—*a*–*b*). The estimate of *K_d_* from the in situ method represents the fractional rate of degradation of fraction *b*; CPD = crude protein degradability at 48 h of incubation. ^1^ Degradation parameters described according to the model by Ørskov and McDonald [16]. ^2^ Effective CPD was calculated from data assuming the fractional rate of passage (K_p_) to be 0.05/h for forage, as used in the protein evaluation system of Hvelplund and Weisbjerg [17]. Different lower-case superscripts in a column indicate a significant difference (*p* < 0.01).

**Table 5:** Fitting regression models to describe the relationship between crude protein degradability and rate of degradation for cereal and legume fodders collected from Lahore and Rawalpindi.

Regression Model	*p*-Value	R^2^	SE	r
Cereal fodders				
Linear	0.001	0.44	0.088	0.66
Quadratic	0.01	0.46	0.088	
Cubic	0.04	0.47	0.089	
Legume fodders				
Linear	0.001	0.60	0.035	0.78
Quadratic	0.001	0.79	0.027	
Cubic	0.001	0.81	0.027	

R^2^ = coefficient of determination; SE = standard error of estimate; r = correlation.

## Data Availability

The data can be made available from the corresponding author upon reasonable request.
